# Association Between C-Reactive Protein–Triglyceride–Glucose Index and Adverse Prognosis Outcomes in Patients with Acute Myocardial Infarction

**DOI:** 10.3390/jcdd13070298

**Published:** 2026-07-01

**Authors:** Xing-Hong Lin, Xin Xu, Ruo-Nan Xu, Cai-Yun Song, Xue-Cheng Song, Peng-Xiang Wang, Yang He, Meng-Die Xia, Rui Feng, Cheng-Gong Sun, Yong-Ming He

**Affiliations:** 1 Division of Cardiology, The First Affiliated Hospital of Soochow University, Suzhou 215006, China; linxinghong2023@163.com (X.-H.L.); hsu18752789840@163.com (X.X.); songcaiyun2026@163.com (C.-Y.S.); sxcphd@163.com (X.-C.S.); wangpengxiang0913@163.com (P.-X.W.); zephyrxmd@163.com (M.-D.X.); frmedicine@163.com (R.F.); 2 Division of Cardiology, Shenzhen Nanshan District Peoples Hospital, Shenzhen 518000, China; xuruonan19982021@163.com; 3 Division of Cardiology, The Hospital of Sihong, Suqian 223900, China; 4 Division of Cardiology, The Third the People’s Hospital of Bengbu, Bengbu 233000, China; 5 Division of Cardiology, The Xiang Cheng People’s Hospital of Suzhou, Suzhou 215000, China; 6 Division of Emergency Medicine, The First Affiliated Hospital of Soochow University, Suzhou 215000, China; heyang0531@suda.edu.cn; 7 Division of Cardiology, Tongren Hospital, Shanghai Jiao Tong University School of Medicine, Shanghai 200336, China

**Keywords:** C-reactive protein–triglyceride–glucose index, insulin resistance, adverse outcomes

## Abstract

Background: The C-reactive protein–triglyceride–glucose index (CTI) integrates inflammation and insulin resistance, but its association with adverse outcomes in acute myocardial infarction (AMI) patients remains unclear. This study aimed to investigate the association between CTI and adverse outcomes, including major adverse cardiac events (MACEs), all-cause death, cardiac death, and myocardial infarction in AMI patients. Methods: The CTI of patients with acute myocardial infarction was calculated and divided into three groups according to its tertiles. Multivariable Cox regression, restricted cubic spline analysis, survival analysis, and correlation heat map analysis were used to explore the association between the CTI and adverse outcomes such as major adverse cardiac events (MACEs), cardiac death, all-cause death and myocardial infarction in patients with acute myocardial infarction. Results: A total of 965 patients were enrolled in this study. In the adjusted Model 3, CTI remained significantly associated with myocardial infarction (Q3 vs. Q1: HR = 2.40, 95% CI: 1.37–4.17, *p* = 0.001; *p* for trend = 0.001). However, the associations with MACEs (HR = 1.29, *p* = 0.161), all-cause death (HR = 1.28, *p* = 0.337), and cardiac death (HR = 1.78, *p* = 0.221) were not statistically significant after full adjustment. Restricted cubic spline analysis identified a threshold effect for myocardial infarction at CTI > 5.92, above which the risk increased 5.61-fold (HR = 5.61, 95% CI: 2.94–10.71, *p* < 0.001). The Kaplan–Meier survival curve showed that the higher the CTI tertiles, the higher the incidence of adverse outcomes. Conclusions: Elevated CTI levels were independently associated with an increased risk of myocardial infarction in AMI patients.

## 1. Introduction

In China, more than 40% of deaths are due to cardiovascular issues, driven by an aging population and common cardiometabolic risk factors [1]. Despite significant improvements in revascularization strategies and optimal medical therapy, the incidence of adverse cardiovascular outcomes remains high, especially in those with multiple cardiometabolic abnormalities. With the increasing incidence of cardiovascular and metabolic factors worldwide, the prevalence of related cardiovascular diseases is also increasing, posing great challenges to the prevention of cardiovascular diseases and death [2,3].

In recent years, inflammation and metabolic abnormalities have been considered important pathological mechanisms of cardiovascular diseases [4]. High-sensitivity C-reactive protein (Hs-CRP) is a sensitive inflammatory marker closely associated with the occurrence of cardiovascular events. Studies have shown that elevated CRP levels are linked to increased risks of atherosclerosis, myocardial infarction, and other cardiovascular events [5,6]. Insulin resistance indicates that the physiological efficacy of insulin is weakened, which is a common pathological mechanism of various metabolic disorders and is closely related to the occurrence and progression of atherosclerosis [7]. The triglyceride–glucose (TyG) is an indicator of insulin resistance and shows potential values in assessing the risk of metabolic syndrome and cardiovascular disease [8]. Insulin resistance exacerbates the inflammatory response, and inflammation leads to insulin resistance, both of which are detrimental to patient prognosis. However, a single indicator is not sufficient to reflect this interaction, so it is particularly important to develop a composite index to reflect their joint predictive role in adverse outcomes. The C-reactive protein–triglyceride–glucose index (CTI) comprehensively reflects the status of insulin resistance and inflammation in patients and has been widely used in clinical studies, including cancer-related consumption syndrome, population-level cancer mortality, and coronary heart disease development [9,10,11]. However, the relationship between the CTI and adverse outcomes in patients with acute myocardial infarction has not been adequately and systematically validated through clinical studies. Therefore, this study aimed to investigate the association between the CTI and adverse outcomes in AMI patients.

## 2. Materials and Methods

### 2.1. Study Design and Study Population

This study retrospectively included consecutive patients admitted during the period from 1 January 2012 to 30 September 2015. All these patients were diagnosed with acute myocardial infarction and underwent Coronary Angiography (CAG examination). Exclusion criteria included: (1) valvular heart disease (moderate to severe valvular stenosis or insufficiency); (2) poor CAG images and not receiving CAG; (3) prior stenting; (4) chronic total occlusion; (5) prior myocardial infarction; (6) repeat hospitalizations, a second or subsequent hospitalization event in the same patient due to “acute myocardial infarction” or “post-procedural complications related to percutaneous coronary intervention “ within the study enrollment time window; (7) lost to follow-up; and (8) incomplete data of TyG and hs-CRP. The study followed the guidelines of the Declaration of Helsinki for human research and was sanctioned by the Institutional Review Board of Soochow University (No. 2020089).

### 2.2. Calculation of the CTI

The laboratory analysis utilized blood sample data collected within 24 h of patient admission, with glucose, lipid metabolism parameters, and liver enzyme parameters measured under fasting conditions (with at least 8 h between the last meal and blood sampling). The CTI consists of CRP and TyG. The TyG index consists of triglycerides (TG) and fasting blood glucose (FBG). The CTI is calculated according to this formula [12]:

CTI  =  0.412  ×  ln (Hs  −  CRP [mg/L])  +  ln (TG [mg/dL] × FBG [mg/dL])

### 2.3. Clinical Data Collection and Definitions

Data on demographics, lab results, and additional details were gathered from the electronic medical records system. The formula for BMI was weight in kilograms divided by height in meters squared. A systolic blood pressure of at least 140 mmHg and/or a diastolic blood pressure of at least 90 mmHg, or a diagnosis based on patient self-report or current use of antihypertensive medication, defined hypertension [13]. Diabetes was diagnosed based on prior diagnosis and receiving hypoglycemic medication or following the recommendations of the American Diabetes Association [14]. The diagnosis of AMI was based on the third universal definition of myocardial infarction [15].

### 2.4. Follow-Up and Endpoint Events

The primary endpoint of this study was major adverse cardiac events (MACEs), defined as a composite of myocardial infarction, cardiac death, and ischemia-driven revascularization; secondary endpoints were all-cause death, cardiac death, and myocardial infarction [16]. All patients were followed up until the date of these endpoints or at four years, whichever came first.

### 2.5. Statistical Analysis

The participants were categorized into three groups according to the tertile of the CTI. Continuous variables with a normal distribution were presented as means with standard deviations, whereas those not normally distributed were shown as medians with interquartile ranges (IQRs). Counts (percentages) were used to express categorical variables. For continuous variables, normally distributed data were analyzed using ANOVA, and non-normally distributed data were assessed with the Kruskal–Wallis test. Categorical variables were analyzed using the chi-squared test. Regarding data integrity, our analysis included only patients with complete data for all key variables, as those with incomplete data were excluded according to the criteria. Therefore, no supplementation method was required. Correlation heat maps were used to analyze correlations between independent variables and between independent variables and adverse outcomes. Correlation heat maps were used to analyze correlations between independent variables and between independent variables and adverse outcomes. To explore the hazard ratios (HRs) and 95% confidence intervals (CIs) of the relationship between the CTI and adverse prognostic outcomes, a multivariable Cox proportional hazards regression model was established. Model 1 remained unadjusted; Model 2 accounted for age and sex; Model 3 incorporated all variables from Model 2 along with primary hypertension, type 2 diabetes, BMI, smoking, alcohol consumption, left ventricular ejection fraction (LVEF), creatinine, and types of myocardial infarction (STEMI or non-STEMI). Restricted cubic spline analysis was used to investigate the linear or nonlinear association between the CTI and adverse outcomes. Event-free survival curves were generated using the Kaplan–Meier method, and survival rates among the tertiles were compared using the log-rank test. In addition, subgroup analysis was performed to determine whether the relationship between the CTI and adverse outcomes remained across different groups. The potential nonlinear correlations between the CTI level and adverse outcomes were assessed on a continuous scale with the restricted cubic spline (RCS) method. Three equally spaced knots were set at the 10th, 50th, and 90th percentiles. We performed two-piecewise linear regression using the segmented package in R. The initial breakpoint was estimated by the Davies test and then refined by an iterative algorithm. The inflection point reported was the point that maximized the model’s likelihood. Using the R software (version 4.4.3, the R Foundation for Statistical Computing, Vienna, Austria), statistical analysis was carried out, with a two-tailed *p* < 0.05 marking a statistically significant difference.

## 3. Results

### 3.1. Baseline Characteristics

**Figure 1:**
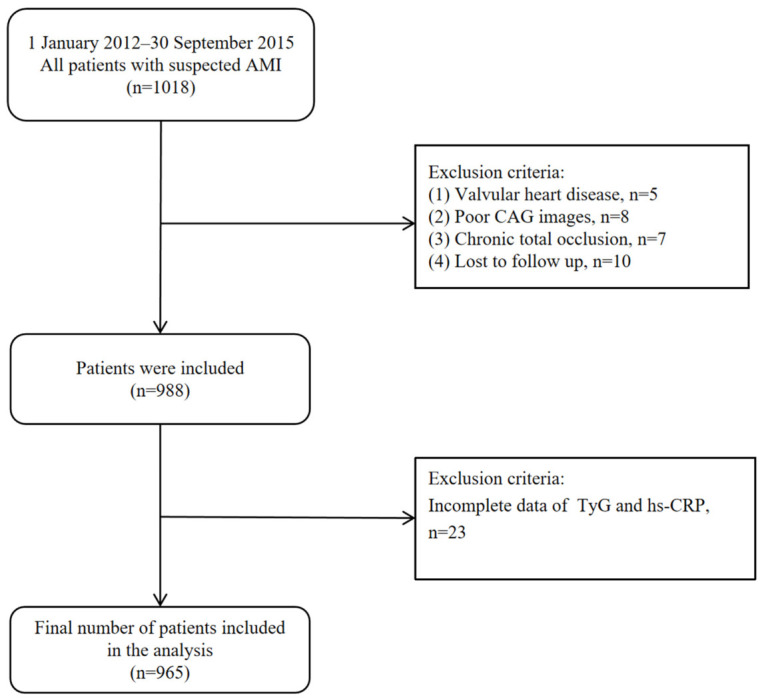
Flowchart of patient enrolment. CAG = coronary angiography; TyG = triglyceride–glucose; Hs-CRP = high-sensitivity C-reactive protein.

As shown in Figure 1, 1018 patients were identified for analysis, with 53 excluded, resulting in 965 meeting the inclusion criteria. Table 1 shows the baseline characteristics stratified by the tertile of the CTI value. The average age was 66 years, and most of the participants in this cohort were male. Notably, individuals with higher CTI values tended to be older and had higher levels of BMI, glucose, TyG, LDL-C, Hs-CRP, and urea. In terms of coronary anatomical characteristics, the proportions of individuals with heavy calcification of the lesion were greater in the third-tertile group. Furthermore, they exhibited a higher prevalence of hypertension and diabetes mellitus.

### 3.2. Correlation Analysis Between Cardiovascular Risk Factors and Adverse Outcomes

**Figure 2:**
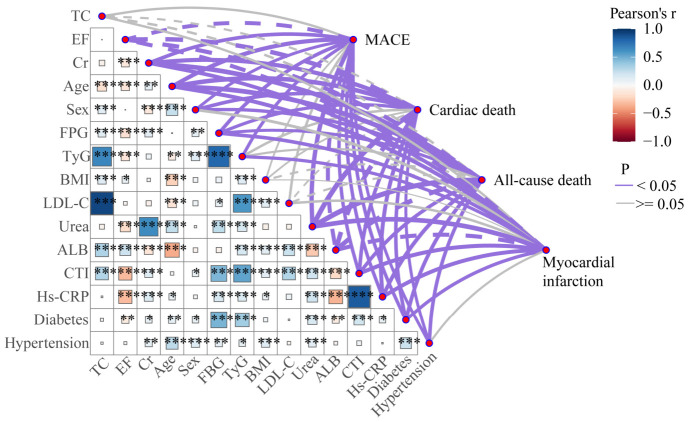
Pearson correlation between variables and adverse outcomes. Within the plot, the triangular portion highlights the correlations between variables. Dark blue squares denote positive correlations, and orange squares denote negative correlations. The size of each square corresponds to the absolute value of Pearson’s correlation coefficient. The higher-right section depicts the correlation between variables and adverse outcomes. Purple indicates significant correlation (*p* < 0.05), gray indicates non-significant correlation (*p* ≥ 0.05), thicker lines indicate larger absolute values of the correlation coefficient, solid lines indicate positive correlation, dashed lines indicate negative correlation. Significance levels are denoted as follows: * *p* < 0.05, ** *p* < 0.01, *** *p* < 0.001. TC = total cholesterol; EF = ejection Fraction; Cr = serum creatinine; FPG = fasting plasma glucose; CTI = C-reactive protein–triglyceride–glucose index; Hs-CRP = high-sensitivity C-reactive protein; LDL-C = low-density lipoprotein–cholesterol; ALB = albumin; TyG = triglyceride–glucose; and BMI = body mass index.

Figure 2 shows the correlations between variables and between variables and adverse outcomes. In the triangular section, darker blue and red-orange represent strong positive correlations and negative correlations between variables, respectively. For example, the CTI showed positive correlations with C-reactive protein, TyG, LDL-C, fasting blood glucose, and urea, suggesting potential links to metabolic disorders, renal impairment, and systemic inflammation. Conversely, the CTI exhibited negative correlations with EF and albumin, indicating that elevated CTI levels may reflect poor cardiac function and nutritional status. These findings demonstrated that the CTI demonstrated consistent variations with multiple conventional mortality-related factors, thereby enhancing its biological validity as a clinically significant prognostic biomarker. The right line shows the correlation between variables and adverse outcomes. The purple and solid lines indicate that all adverse outcomes were positively correlated with the CTI (*p* <0.05).

### 3.3. Associations Between the CTI and Adverse Outcomes

As shown in Table 2, in Model 1, the CTI, as a continuous variable, was significantly associated with all four endpoints: MACEs (HR = 1.71, 95% CI: 1.19–2.45, *p* = 0.004), all-cause death (HR = 2.27, 95% CI: 1.39–3.70, *p* = 0.001), cardiac death (HR = 3.16, 95% CI: 1.61–6.21, *p* < 0.001), and myocardial infarction (HR = 3.47, 95% CI: 2.05–5.88, *p* < 0.001). In Model 2, the associations remained largely unchanged. However, in the fully adjusted Model 3, the associations with MACEs, all-cause death, and cardiac death were substantially attenuated and no longer statistically significant: the HRs for Q3 versus Q1 were 1.29 (95% CI: 0.90–1.84, *p* = 0.161), 1.28 (95% CI: 0.76–2.14, *p* = 0.337), and 1.78 (95% CI: 0.78–2.57, *p* = 0.221), with all *p* values for trends > 0.05. In contrast, the association between the CTI and myocardial infarction remained statistically significant under the same full adjustment: the HR for Q3 versus Q1 was 2.40 (95% CI: 1.37–4.17, *p* = 0.001), with a *p* value trend of 0.001.

### 3.4. RCS Analysis and Threshold Effect Analysis Investigating the Relationship Between the CTI and Adverse Outcomes

**Figure 3:**
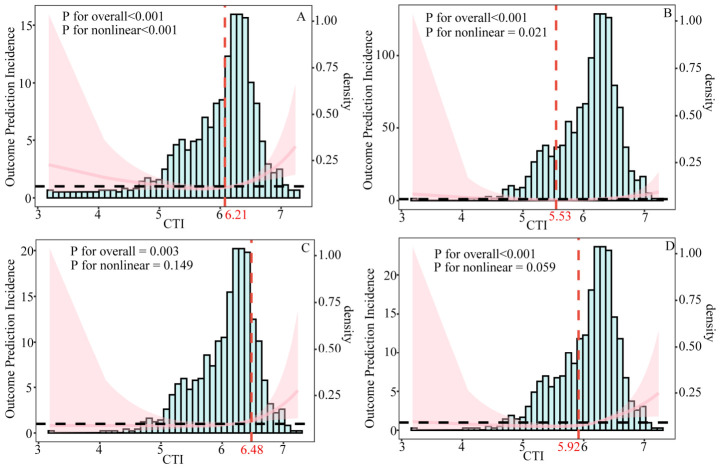
RSC showing the connection between the CTI and adverse outcomes. (**A**) MACEs; (**B**) cardiac death; (**C**) all-cause death; (**D**) myocardial infarction. CTI = C-reactive protein–triglyceride–glucose index; MACEs = major adverse cardiac events. The pink shaded area represents the 95% confidence interval. The pink solid line shows the trend of the estimated hazard ratio (HR) with changes in CTI. The black dashed line indicates the horizontal reference line HR = 1.

To further characterize the dose–response relationship between the CTI and adverse outcomes, we performed RCS analysis for each endpoint separately. This study found a nonlinear relationship between the CTI and MACEs and cardiac death (*p* for nonlinear < 0.05) (Figure 3A,B), whereas it showed a linear relationship with all-cause death and myocardial infarction (Figure 3C,D). Two-piecewise linear regression showed that the inflection points were 6.21 for MACEs, 5.53 for cardiac death, 6.48 for all-cause death and 5.92 for myocardial infarction. When the CTI exceeded these thresholds, the risks increased significantly across all endpoints. Among these, the threshold for myocardial infarction was 5.92; exceeding this threshold increased the risk by 5.61-fold (HR = 5.61,95% CI: 2.94–10.71, *p* <0.001), suggesting that the CTI may have a clinical threshold for identifying patients at elevated risk of ischemic recurrence (Table 3).

### 3.5. Kaplan–Meier Survival Curves and Subgroup Analyses Showing the Relationship Between CTI and Adverse Outcomes

**Figure 4:**
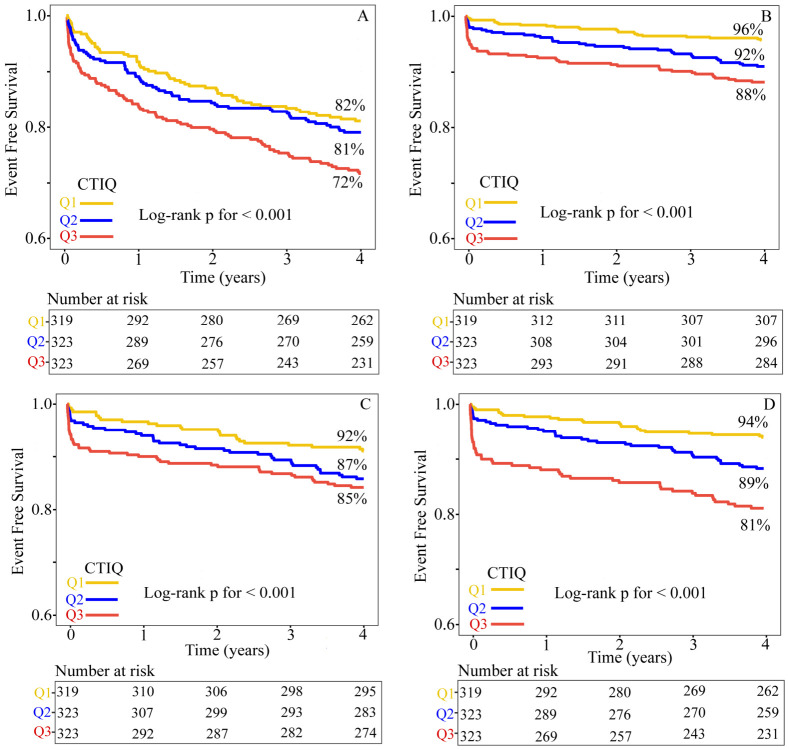
Kaplan–Meier curves for adverse outcomes at 4 years according to CTI tertiles. (**A**) MACEs; (**B**) cardiac death; (**C**) all-cause death; (**D**) myocardial infarction. CTI = C-reactive protein–triglyceride–glucose index; MACEs = major adverse cardiac events.

**Figure 5:**
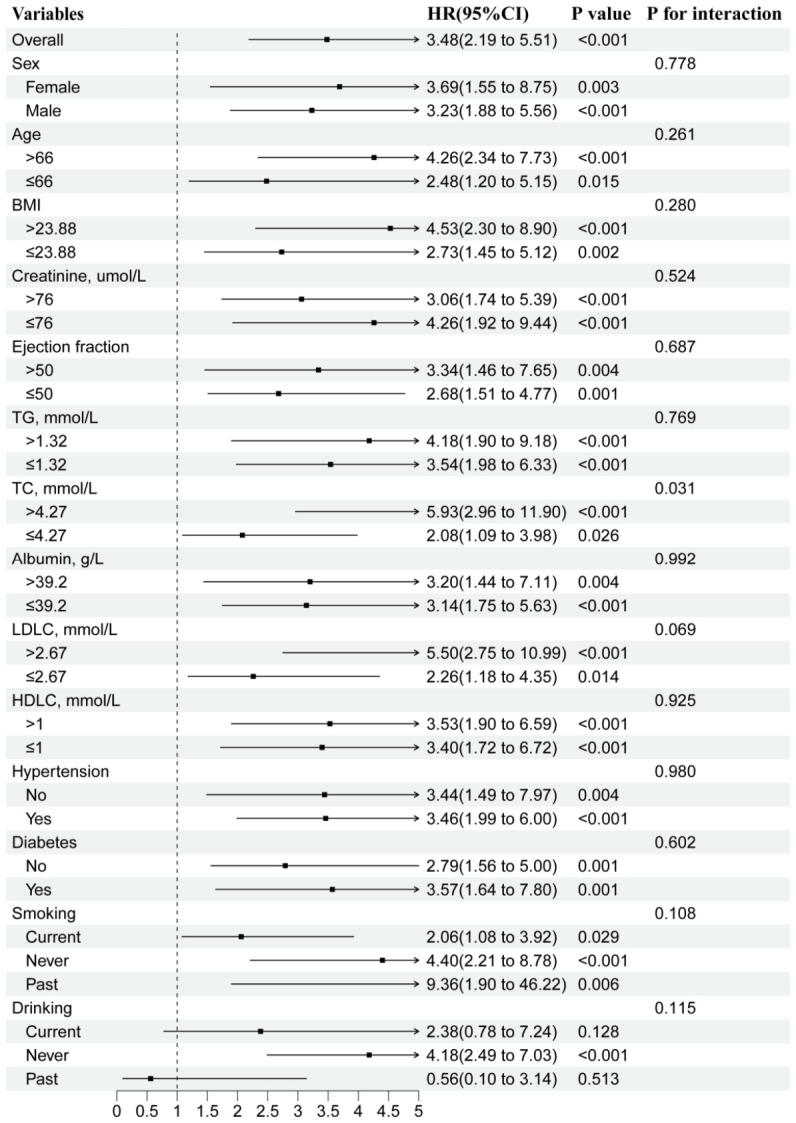
Subgroup analyses of the associations of the CTI with myocardial infarction across the subgroups of covariates, dichotomously or medially. CTI = C-reactive protein–triglyceride–glucose index; BMI = body mass index; TG = triglyceride; TC = total cholesterol; LDL-C = low-density lipoprotein–cholesterol; HDL-C = high-density lipoprotein–cholesterol; and MACEs = major adverse cardiac events.

The Kaplan–Meier (K-M) survival curve showed that the higher the CTI values, the higher the incidences of MACEs, cardiac death, all-cause death and myocardial infarction (Figure 4). As shown in Figure 5, subgroup analysis revealed the consistent associations of the CTI with the adverse outcomes, without significant interactions across subgroups (all *p* values for interaction > 0.05), indicating that the association between the CTI and myocardial infarction was applicable across different patient strata. Subgroup analyses for other endpoints are presented in the Supplementary Materials (Figures S1–S3).

## 4. Discussion

The key findings of the current study were as follows: (1) After comprehensive adjustment for confounders, the CTI remained significantly associated with myocardial infarction (Q3 vs. Q1: HR = 2.40, 95% CI:1.37–4.17, *p* = 0.001); (2) in contrast, the associations with MACEs, all-cause death, and cardiac death were substantially attenuated and no longer statistically significant after the same adjustment; (3) RCS analysis identified a threshold effect for myocardial infarction at CTI > 5.92, above which the risk increased 5.61-fold.

The association between the CTI and myocardial infarction, even after full adjustment, was biologically plausible. Myocardial infarction is predominantly driven by plaque instability and thrombotic occlusion, processes directly promoted by systemic inflammation and insulin resistance. CRP induces endothelial dysfunction, enhances oxidized lipoprotein deposition, and facilitates leukocyte infiltration, while insulin resistance impairs nitric oxide bioavailability, promotes a pro-thrombotic state, and exacerbates lipotoxicity. By integrating inflammation and insulin resistance, the CTI captures a dual pathophysiological process. The exact mechanism by which the CTI increases the risk of myocardial infarction remains to be elucidated. However, the following factors may provide potential explanations for this association. Ischemic injury and chronic inflammation have been demonstrated to impair vascular endothelial integrity, reduce nitric oxide utilization efficiency, disrupt normal coagulation mechanisms, and accelerate atherosclerosis progression [17]. These factors, in turn, have been shown to significantly increase the risk of myocardial infarction [18,19]. Furthermore, inflammation amplifies systemic inflammatory responses through tissue-derived mediators, thereby exacerbating insulin resistance. This mutually reinforcing cycle further elevates myocardial infarction risk [20]. Concurrently, inflammation and insulin resistance destabilize atherosclerotic plaques, increasing the risk of rupture and subsequent thrombosis, which promotes myocardial infarction development [21]. In summary, patients with insulin resistance and inflammation often present with comorbid conditions such as hypertension, diabetes, and obesity, all of which are recognized as major risk factors for myocardial infarction [22,23]. Our findings were consistent with and extended those of Meng et al., who demonstrated in a large prospective cohort (n = 94,509) that each 1-unit increment in the CTI was associated with a 29% increase in the risk of myocardial infarction (HR = 1.29, 95% CI: 1.74–2.40) [24].

In contrast to its robust association with myocardial infarction, the CTI’s associations with MACEs, all-cause death, and cardiac death were attenuated and became non-significant after adjustment for LVEF, creatinine, and types of myocardial infarction (STEMI or non-STEMI), suggesting that these associations were largely explained by cardiac and renal function, as well as disease severity. Baseline LVEF decreased significantly across CTI tertiles (56% vs. 48% vs. 45%, *p* < 0.001), demonstrating an association between the CTI and cardiac function. This correlation suggested that the inflammatory–metabolic burden reflected by the CTI was linked to cardiac dysfunction.

The identification of a threshold for myocardial infarction at a CTI > 5.92 has potential clinical implications. Patients exceeding this threshold had a more than 5-fold higher risk of myocardial infarction (HR = 5.61, 95% CI: 2.94–10.71, *p* < 0.001). This finding suggested the presence of a possible risk threshold. However, this inflection point was derived from exploratory analysis, which was limited by sample size and event count, and relied on a single baseline measurement, thus failing to reflect the dynamic changes in the CTI. Moreover, these findings were specific to the study population and may require validation in independent, multicenter, large-sample cohorts in future studies.

## 5. Limitations

There are several limitations to this study. Firstly, this was a single-center retrospective cohort study, so there was always the potential for selection bias. Secondly, the relatively small sample size meant the exact CTI threshold and the generalizability of the conclusions still required further confirmation in a multicenter study. Finally, the CTI and its components were measured only at baseline; dynamic changes over time were not captured.

## 6. Conclusions

Elevated CTI levels were independently associated with an increased risk of myocardial infarction in AMI patients.

## Figures and Tables

**Table 1:** Characteristics of the study population based on the tertile of CTI.

Variables	Overall	Q1	Q2	Q3	*p* Value
	*N* = 965	319	323	323	
Age (y)	66.00 (57.00, 74.00)	65.00 (57.00, 72.00)	67.00 (58.00, 76.00)	66.00 (57.00, 73.00)	0.020
Sex (n, %)					0.015
Male	765.00 (79.27%)	263.00 (82.45%)	263.00 (81.42%)	239.00 (73.99%)	
Female	200.00 (20.73%)	56.00 (17.55%)	60.00 (18.58%)	84.00 (26.01%)	
Drinking (n, %)					0.997
Never	712.00 (73.78%)	236.00 (73.98%)	240.00 (74.30%)	236.00 (73.07%)	
Past	33.00 (3.42%)	11.00 (3.45%)	11.00 (3.41%)	11.00 (3.41%)	
Current	220.00 (22.80%)	72.00 (22.57%)	72.00 (22.29%)	76.00 (23.52%)	
Smoking (n, %)					0.393
Never	353.00 (36.58%)	104.00 (32.60%)	125.00 (38.70%)	124.00 (38.39%)	
Past	94.00 (25.75%)	30.00 (9.40%)	34.00 (10.53%)	30.00 (9.29%)	
Current	518.00 (53.67%)	185.00 (58.00%)	164.00 (50.77%)	169.00 (52.32%)	
Stelev (n, %)					<0.001
non-STEMI	479.00 (49.64%)	191.00 (59.87%)	166.00 (51.39%)	122.00 (37.77%)	
STEMI	486.00 (50.36%)	128.00 (40.13%)	157.00 (48.61%)	201.00 (62.23%)	
Heavy calcification (n, %)	114.00 (11.81%)	28.00 (8.80%)	38.00 (11.76%)	48.00 (14.86%)	0.058
Hypertension (n, %)	639.00 (66.22%)	210.00 (65.83%)	206.00 (63.78%)	223.00 (69.04%)	0.362
Diabetes mellitus (n, %)	228.00 (23.63%)	54.00 (16.93%)	58.00 (17.96%)	116.00 (35.91%)	<0.001
BMI (kg/m^2^)	23.88 (22.02,26.12)	23.66 (21.64, 25.71)	24.09 (21.88, 26.12)	24.46 (22.34, 26.81)	0.006
LDL-C (mmol/L)	2.67 (2.15, 3.35)	2.54 (2.10, 3.04)	2.48 (2.10, 3.12)	3.19 (2.51, 3.77)	<0.001
Albumin (g/L)	39.20 (36.30, 41.80)	40.20 (38.00, 42.90)	37.90 (35.60, 40.60)	38.90 (35.80, 41.60)	<0.001
Creatinine (umol/L)	76.00 (65.00,91.00)	75.00 (64.00, 88.00)	78.00 (65.00, 93.00)	77.00 (65.00, 95.00)	0.044
LVEF (%)	50.00 (43.00, 61.00)	56.00 (46.00, 65.00)	48.00 (43.00, 61.00)	45.00 (40.00, 56.00)	<0.001
Hs-CRP (mg/L)	10.40 (3.33, 13.36)	1.98 (1.01, 3.67)	11.07 (7.76, 13.18)	13.48 (12.05, 14.50)	<0.001
TC (mmol/L)	4.27 (3.64, 5.00)	4.10 (3.49, 4.65)	3.96 (3.43, 4.72)	4.81 (4.08, 5.54)	<0.001
TG (mmol/L)	1.32 (0.92, 1.89)	1.32 (0.91, 1.94)	1.22 (0.83, 1.80)	1.22 (0.83, 1.80)	<0.001
Blood glucose (mmol/L)	5.65 (5.04, 7.04)	5.31 (4.72, 5.98)	5.41 (4.86, 6.07)	7.04 (5.87, 9.72)	<0.001
Blood Urea (mmol/L)	5.20 (4.20, 6.80)	5.00 (4.20, 6.10)	5.10 (4.10, 6.50)	5.60 (4.40, 7.70)	<0.001

Notes: CTI = C-reactive protein–triglyceride–glucose index; STEMI = ST-segment elevation myocardial infarction; BMI = body mass index; LDL-C = low-density lipoprotein–cholesterol; LVEF = left ventricular ejection fraction; Hs-CRP = high-sensitivity C-reactive protein; TC = total cholesterol; and TG = triglyceride. Categorical variables were expressed as n (%), and continuous variables were represented by median (interquartile range).

**Table 2:** Multivariate Cox regression analysis of CTI with clinical outcomes.

Characteristic	Model 1		Model 2		Model 3	
	HR (95% CI)	*p* Value	HR (95% CI)	*p* Value	HR (95% CI)	*p* Value
**MACE**						
Continuous	1.71 (1.19, 2.45)	0.004	1.71 (1.17, 2.50)	0.005	1.28 (0.87, 1.87)	0.205
Categorical						
Q1	1 (Ref)		1 (Ref)		1 (Ref)	
Q2	1.13 (0.79, 1.61)	0.501	1.03 (0.72, 1.47)	0.860	0.92 (0.64, 1.32)	0.656
Q3	1.73 (1.25, 2.41)	0.001	1.73 (1.24, 2.41)	0.001	1.29 (0.90, 1.84)	0.161
*p* for trend		0.001		0.001		0.140
**All-cause death**						
Continuous	2.27 (1.39, 3.70)	0.001	2.22 (1.30, 3.81)	0.004	1.47 (0.86, 2.51)	0.157
Categorical						
Q1	1 (Ref)		1 (Ref)		1 (Ref)	
Q2	1.69 (1.02, 2.79)	0.042	1.31 (0.80, 2.16)	0.289	1.16 (0.69, 1.95)	0.568
Q3	2.14 (1.32, 3.49)	0.002	1.97 (1.21, 3.20)	0.006	1.28 (0.76, 2.14)	0.337
*p* for trend		0.002		0.005		0.337
**Cardiac death**						
Continuous	3.16 (1.61, 6.21)	<0.001	3.13 (1.51, 6.49)	0.002	1.83 (0.853, 3.946)	0.119
Categorical						
Q1	1 (Ref)		1 (Ref)		1 (Ref)	
Q2	2.27 (1.15, 4.46)	0.018	1.74 (0.88, 3.44)	0.109	1.41 (0.67, 2.81)	0.324
Q3	3.40 (1.78, 6.48)	<0.001	3.07 (1.62, 5.85)	<0.001	1.78 (0.78, 2.57)	0.221
*p* for trend		<0.001		<0.001		0.089
**Myocardial infarction**						
Continuous	3.47 (2.05, 5.88)	<0.001	3.57 (2.05, 6.23)	<0.001	2.44 (1.37, 4.34)	0.002
Categorical						
Q1	1 (Ref)		1 (Ref)		1 (Ref)	
Q2	1.82 (1.04, 3.18)	0.036	1.63 (0.93, 2.83)	0.086	1.39 (0.79, 2.43)	0.245
Q3	3.51 (2.11, 5.85)	<0.001	3.48 (2.09, 5.79)	<0.001	2.40 (1.37, 4.17)	0.001
*p* for trend		<0.001		<0.001		0.001

Notes: CTI = C-reactive protein–triglyceride–glucose index; MACEs = major adverse cardiac events; BMI = body mass index; HR = hazard ratios; and CI = confidence interval. Model 1 remained unadjusted; Model 2 accounted for age and sex; Model 3 incorporated all variables from Model 2 along with primary hypertension, type 2 diabetes, BMI, smoking, alcohol consumption, LVEF, creatinine, and types of myocardial infarction (STEMI or non-STEMI).

**Table 3:** Threshold effect analysis of CTI on adverse outcomes.

Characteristic	HR (95% CI)	*p*-Value
**MACE**		
Fitting by the standard linear model		
Fitting by the two-piecewise linear model		
Inflection point	6.21	
CTI < 6.21	0.97 (0.65, 1.45)	0.879
CTI ≥ 6.21	5.27 (2.74, 10.14)	<0.001
*p* for log-likelihood ratio		<0.001
**Cardiac death**		
Fitting by the standard linear model		
Fitting by the two-piecewise linear model		
Inflection point	5.53	
CTI < 5.53	0.42 (0.11–1.54)	0.189
CTI ≥ 5.53	4.86 (2.56–9.21)	<0.001
*p* for log-likelihood ratio		0.021
**All-cause death**		
Fitting by the standard linear model		
Fitting by the two-piecewise linear model		
Inflection point	6.48	
CTI < 6.48	1.43 (0.84, 2.42)	0.200
CTI ≥ 6.48	7.78 (1.93, 31.42)	0.004
*p* for log-likelihood ratio		0.060
**Myocardial infarction**		
Fitting by the standard linear model		
Fitting by the two-piecewise linear model		
Inflection point	5.92	
CTI < 5.92	1.29 (0.49, 3.43)	0.606
CTI ≥ 5.92	5.61 (2.94, 10.71)	<0.001
*p* for log-likelihood ratio		0.052

## Data Availability

The original contributions presented in this study are included in the article; further inquiries can be directed to the corresponding author.

## Supplementary Materials

The following supporting information can be downloaded at: https://www.mdpi.com/article/10.3390/jcdd13070298/s1, Figure S1: Subgroup analyses of the associations of the CTI with all-cause death across the subgroups of covariates, dichotomously or medially. CTI, C-reactive protein-triglyceride-glucose index; BMI, body mass index; TG, triglycerides; TC, total cholesterol; LDL-C, low density lipoprotein-cholesterol; and HDL-C, high density lipoprotein cholesterol;. Figure S2: Subgroup analyses of the associations of the CTI with cardiac death across the subgroups of covariates, dichotomously or medially. CTI, C-reactive protein-triglyceride-glucose index; BMI, body mass index; TG, triglycerides; TC, total cholesterol; LDL-C, low density lipoprotein-cholesterol; and HDL-C, high density lipoprotein cholesterol; Figure S3: Subgroup analyses of the associations of the CTI with MACE across the subgroups of covariates, dichotomously or medially. CTI, C-reactive protein-triglyceride-glucose index; BMI, body mass index; TG, triglycerides; TC, total cholesterol; LDL-C, low density lipoprotein-cholesterol; and HDL-C, high density lipoprotein cholesterol.
