# Supplementary material for: Association Between C-Reactive Protein–Triglyceride–Glucose Index and Adverse Prognosis Outcomes in Patients with Acute Myocardial Infarction
Source: J Cardiovasc Dev Dis. 2026 Jul 1;13(7):298. doi: 10.3390/jcdd13070298 (PMC13411109; doi:10.3390/jcdd13070298)
Supplement: Supplementary file 1 [file jcdd-13-00298-s001.zip › jcdd-4373961-supplementary.pdf]

**Supplementary**

# Association Between C-Reactive Protein–Triglyceride–Glucose Index and Adverse Prognosis Outcomes in Patients with Acute Myocardial Infarction

**Xing-Hong Lin [1,†], Xin Xu [1,†], Ruo-Nan Xu [2], Cai-Yun Song [1,3], Xue-Cheng Song [1,4], Peng-Xiang Wang [1,5], Yang He [6], Meng-Die Xia [1], Rui Feng [1], Cheng-Gong Sun [7,*] and Yong-Ming He [1,*]**

[1] Division of Cardiology, The First Affiliated Hospital of Soochow University, Suzhou 215006, China; linxinghong2023@163.com (X.-H.L.); hsu18752789840@163.com (X.X.); songcaiyun2026@163.com (C.-Y.S.); sxcphd@163.com (X.-C.S.); wangpengxiang0913@163.com (P.-X.W.); zephyrxmd@163.com (M.-D.X.); frmedicine@163.com (R.F.)
[2] Division of Cardiology, Shenzhen Nanshan District Peoples Hospital, Shenzhen 518000, China; xuruonan19982021@163.com
[3] Division of Cardiology, The Hospital of Sihong, Suqian 223900, China
[4] Division of Cardiology, The Third the People's Hospital of Bengbu, Bengbu 233000, China
[5] Division of Cardiology, The Xiang Cheng People's Hospital of Suzhou, Suzhou 215000, China
[6] Division of Emergency Medicine, The First Affiliated Hospital of Soochow University, Suzhou 215000, China; heyang0531@suda.edu.cn
[7] Division of Cardiology, Tongren Hospital, Shanghai Jiao Tong University School of Medicine, Shanghai 200336, China
* Correspondence: scg221002@126.com (C.-G.S.); heyongming@suda.edu.cn (Y.-M.H.)
† These authors contributed equally to this work.

| Variables | HR(95%CI) | P value | P for interaction |
|---|---|---|---|
| Overall | 2.27(1.47 to 3.50) | <0.001 | |
| Sex | | | 0.146 |
| Female | 3.46(1.55 to 7.71) | 0.002 | |
| Male | 1.72(1.04 to 2.86) | 0.035 | |
| Age | | | 0.525 |
| >66 | 2.06(1.25 to 3.39) | 0.005 | |
| ≤66 | 2.89(1.11 to 7.51) | 0.03 | |
| BMI | | | 0.049 |
| >23.88 | 3.97(1.93 to 8.13) | <0.001 | |
| ≤23.88 | 1.61(0.94 to 2.78) | 0.084 | |
| Creatinine, umol/L | | | 0.755 |
| >76 | 2.35(1.40 to 3.94) | 0.001 | |
| ≤76 | 2.02(0.91 to 4.49) | 0.086 | |
| Ejection fraction | | | 0.236 |
| >50 | 1.37(0.70 to 2.67) | 0.355 | |
| ≤50 | 2.33(1.30 to 4.18) | 0.005 | |
| TG, mmol/L | | | 0.918 |
| >1.32 | 2.40(1.12 to 5.18) | 0.025 | |
| ≤1.32 | 2.50(1.46 to 4.31) | 0.001 | |
| TC, mmol/L | | | 0.026 |
| >4.27 | 4.09(2.04 to 8.21) | <0.001 | |
| ≤4.27 | 1.46(0.82 to 2.62) | 0.198 | |
| Albumin, g/L | | | 0.527 |
| >39.2 | 1.59(0.73 to 3.45) | 0.244 | |
| ≤39.2 | 2.14(1.24 to 3.69) | 0.006 | |
| LDLC, mmol/L | | | 0.033 |
| >2.67 | 4.12(2.03 to 8.38) | <0.001 | |
| ≤2.67 | 1.53(0.86 to 2.72) | 0.151 | |
| HDLC, mmol/L | | | 0.659 |
| >1 | 2.48(1.36 to 4.53) | 0.003 | |
| ≤1 | 2.06(1.10 to 3.84) | 0.024 | |
| Hypertension | | | 0.621 |
| No | 1.76(0.67 to 4.61) | 0.249 | |
| Yes | 2.32(1.44 to 3.76) | 0.001 | |
| Diabetes | | | 0.748 |
| No | 1.78(1.02 to 3.10) | 0.041 | |
| Yes | 2.05(1.05 to 4.01) | 0.037 | |
| Smoking | | | 0.879 |
| Current | 1.89(0.94 to 3.81) | 0.074 | |
| Never | 2.40(1.30 to 4.42) | 0.005 | |
| Past | 2.07(0.64 to 6.65) | 0.223 | |
| Drinking | | | 0.070 |
| Current | 3.24(0.62 to 16.95) | 0.163 | |
| Never | 2.50(1.56 to 4.01) | <0.001 | |
| Past | 0.31(0.07 to 1.47) | 0.142 | |

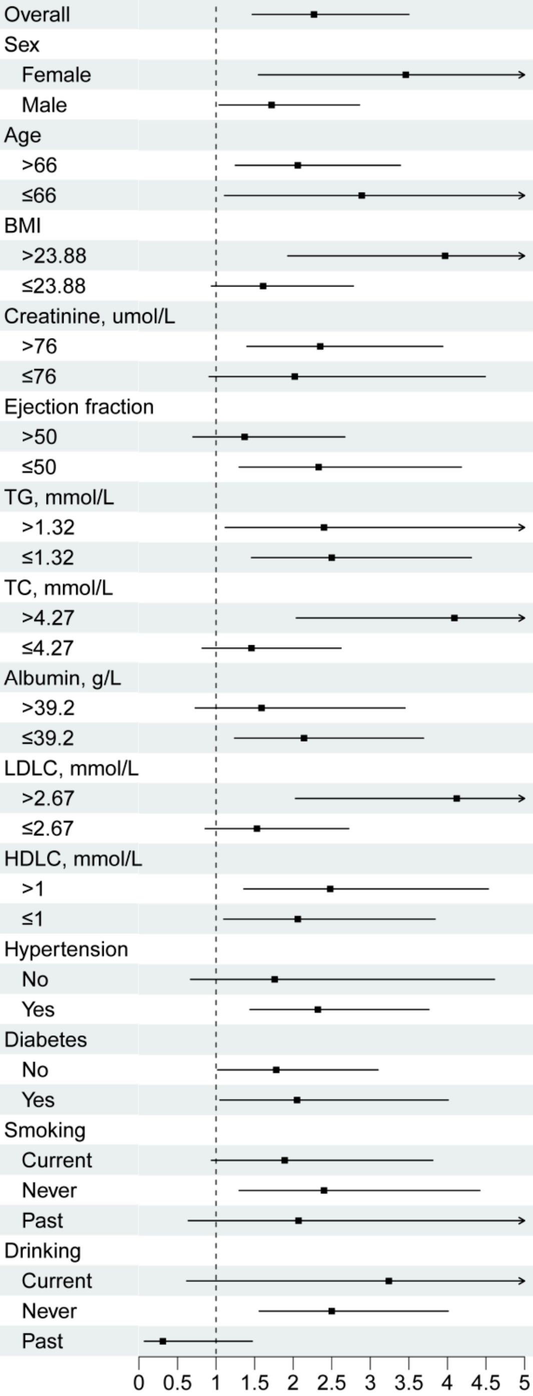


**Figure S1.** Subgroup analyses of the associations of the CTI with all-cause death across the subgroups of covariates, dichotomously or medially. CTI, C-reactive protein-triglyceride-glucose index; BMI, body mass index; TG, triglycerides; TC, total cholesterol; LDL-C, low density lipoprotein-cholesterol; and HDL-C, high density lipoprotein cholesterol.

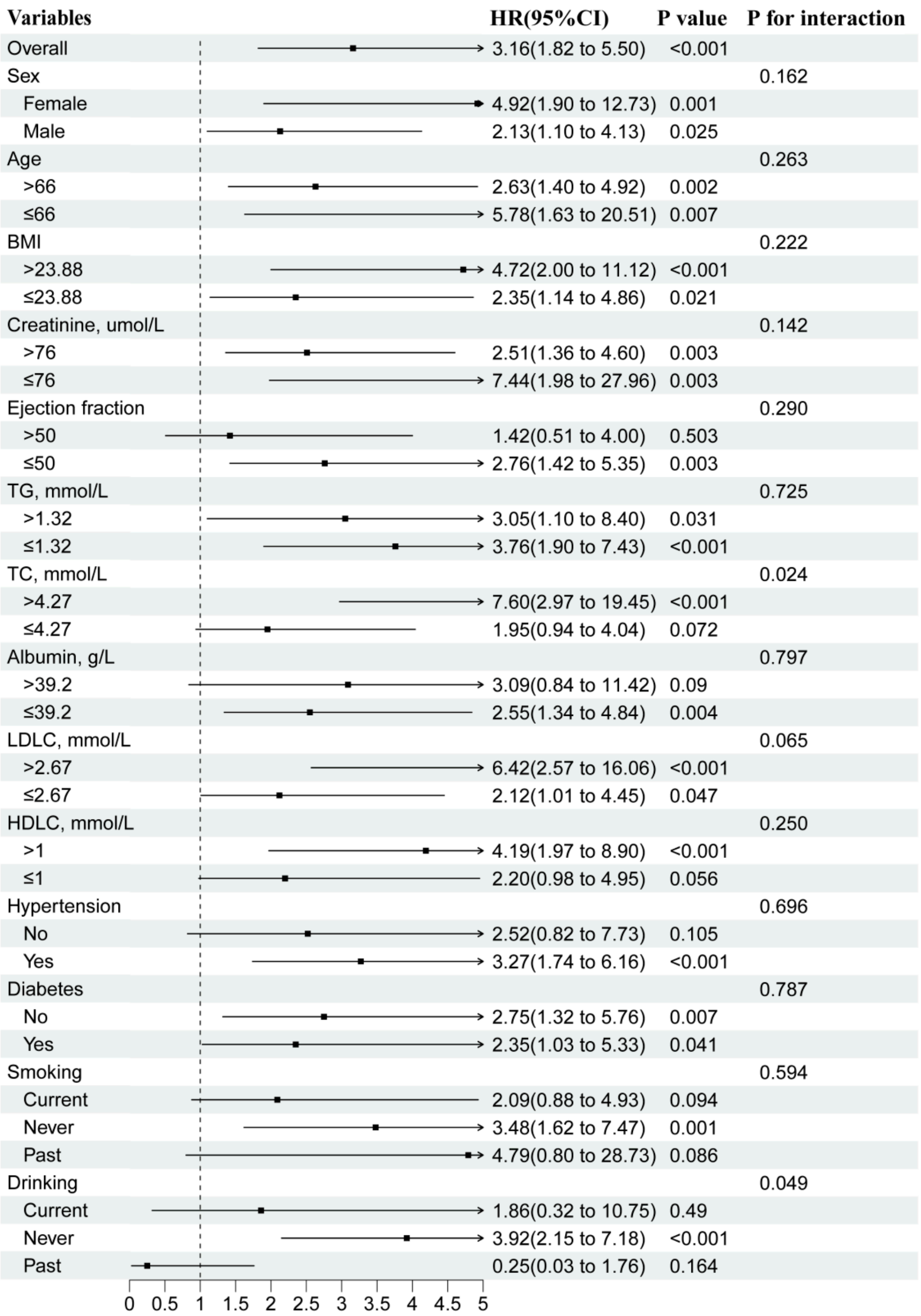


**Figure S2.** Subgroup analyses of the associations of the CTI with cardiac death across the subgroups of covariates, dichotomously or medially. CTI, C-reactive protein-triglyceride-glucose index; BMI, body mass index; TG, triglycerides; TC, total cholesterol; LDL-C, low density lipoprotein-cholesterol; and HDL-C, high density lipoprotein cholesterol.

| Variables | HR(95%CI) | P value | P for interaction |
|---|---|---|---|
| Overall | 1.71(1.27 to 2.31) | <0.001 | |
| Sex | | | 0.681 |
| Female | 1.83(1.04 to 3.25) | 0.038 | |
| Male | 1.60(1.13 to 2.27) | 0.008 | |
| Age | | | 0.086 |
| >66 | 2.14(1.42 to 3.23) | <0.001 | |
| ≤66 | 1.26(0.81 to 1.96) | 0.301 | |
| BMI | | | 0.065 |
| >23.88 | 2.23(1.44 to 3.45) | <0.001 | |
| ≤23.88 | 1.27(0.84 to 1.92) | 0.26 | |
| Creatinine, umol/L | | | 0.433 |
| >76 | 1.89(1.25 to 2.86) | 0.002 | |
| ≤76 | 1.50(0.97 to 2.32) | 0.071 | |
| Ejection fraction | | | 0.644 |
| >50 | 1.62(1.03 to 2.57) | 0.039 | |
| ≤50 | 1.39(0.91 to 2.12) | 0.126 | |
| TG, mmol/L | | | 0.760 |
| >1.32 | 1.87(1.18 to 2.96) | 0.008 | |
| ≤1.32 | 1.68(1.12 to 2.50) | 0.011 | |
| TC, mmol/L | | | 0.200 |
| >4.27 | 2.07(1.33 to 3.22) | 0.001 | |
| ≤4.27 | 1.38(0.90 to 2.12) | 0.137 | |
| Albumin, g/L | | | 0.247 |
| >39.2 | 1.29(0.83 to 2.01) | 0.251 | |
| ≤39.2 | 1.84(1.20 to 2.82) | 0.005 | |
| LDLC, mmol/L | | | 0.168 |
| >2.67 | 2.10(1.35 to 3.26) | 0.001 | |
| ≤2.67 | 1.36(0.89 to 2.09) | 0.156 | |
| HDLC, mmol/L | | | 0.269 |
| >1 | 1.46(0.97 to 2.18) | 0.069 | |
| ≤1 | 2.04(1.31 to 3.19) | 0.002 | |
| Hypertension | | | 0.980 |
| No | 1.67(0.94 to 2.98) | 0.08 | |
| Yes | 1.69(1.19 to 2.40) | 0.003 | |
| Diabetes | | | 0.328 |
| No | 1.40(0.97 to 2.01) | 0.07 | |
| Yes | 1.90(1.12 to 3.24) | 0.017 | |
| Smoking | | | 0.304 |
| Current | 1.28(0.82 to 1.99) | 0.272 | |
| Never | 2.10(1.33 to 3.31) | 0.001 | |
| Past | 1.74(0.75 to 4.06) | 0.2 | |
| Drinking | | | 0.152 |
| Current | 0.98(0.53 to 1.84) | 0.962 | |
| Never | 2.00(1.42 to 2.81) | <0.001 | |
| Past | 1.03(0.20 to 5.29) | 0.972 | |

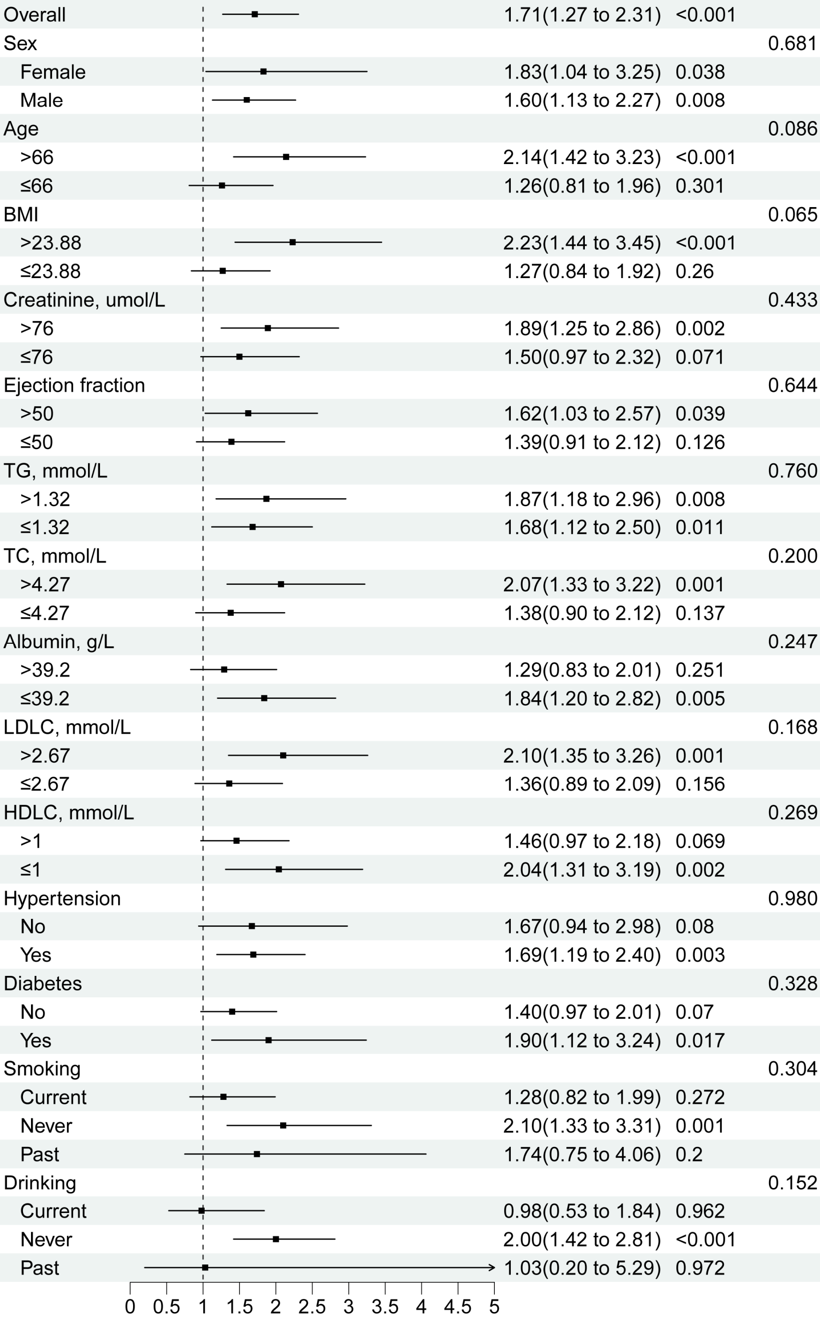


**Figure S3.** Subgroup analyses of the associations of the CTI with MACE across the subgroups of covariates, dichotomously or medially. CTI, C-reactive protein-triglyceride-glucose index; BMI, body mass index; TG, triglycerides; TC, total cholesterol; LDL-C, low density lipoprotein-cholesterol; and HDL-C, high density lipoprotein cholesterol.
